# The association of pregnancy control, emotions, and beliefs with pregnancy desires: A new perspective on pregnancy intentions

**DOI:** 10.1371/journal.pone.0249032

**Published:** 2021-03-23

**Authors:** Meredith G. Manze, Diana R. Romero, Prabal De, Josette Hartnett, Lynn Roberts

**Affiliations:** 1 Department of Community Health and Social Sciences, City University of New York (CUNY) Graduate School of Public Health and Health Policy, New York, New York, United States of America; 2 Economics Program, City University of New York (CUNY) Graduate Center, New York, New York, United States of America; 3 Department of Economics and Business, City College of New York, New York, New York, United States of America; 4 Office of Research and Clinical Trials, Stamford Hospital, Stamford, Connecticut, United States of America; Ordu University, TURKEY

## Abstract

**Context:**

Standard pregnancy intentions measures do not always align with how people approach pregnancy. Studies that have investigated beyond a binary framework found that those with “ambivalent” feelings towards pregnancy are less likely to use contraception consistently, but the reasons for this are unclear. We sought to gain a nuanced understanding of pregnancy desires, and how perceptions about pregnancy are associated with contraceptive use.

**Methods:**

We used non-probability quota sampling based on sex, age, and geographic region for a web-based survey of heterosexual men and women, aged 21–44 years, who could become pregnant/impregnate and were not currently pregnant (n = 1,477; 51% female). The survey was created using unique items informed by recent literature. Bivariate and multivariable analyses explored relationships between various perceptions about pregnancy with pregnancy *desires* categorized as: *wanting a pregnancy*, *not wanting a pregnancy*, and *not trying but would be okay with a pregnancy*. We conducted a sub-group analysis of those who were not trying but would be okay with a pregnancy (n = 460), using descriptive statistics to examine how consistent contraceptive use was associated with emotions and beliefs about avoiding pregnancy.

**Results:**

After adjustment, those who felt that pregnancy may not always be avoidable, but instead determined by fate/higher power, or a natural process that happens when it is meant to, were significantly more likely (aOR: 1.83, 95% CI: 1.05–3.36; aOR: 2.21, 95% CI: 1.29–3.76, respectively) to report not trying but being okay with pregnancy, whereas those with negative feelings about a pregnancy were less likely to feel okay about a pregnancy (aOR: 0.11, 95% CI: 0.08–0.15), versus not wanting a pregnancy. In the sub-group analysis, those who felt pregnancy was determined by fate/higher power or a natural process were more likely to report not using contraception consistently (70%, 68%, respectively).

**Conclusions:**

Those who state they are not trying but would be okay with pregnancy may not use contraception consistently because of beliefs that pregnancy is predetermined. Our findings support less categorical and more multidimensional approaches to measuring fertility intentions, with important implications for reproductive health service provision.

## Introduction

Demographers and other public health researchers have measured pregnancy intentions for nearly 80 years in order to understand fertility-related behaviors and trends [1]. Over time, the definition of pregnancy intentions has been refined as our understanding of sexual behavior and family planning has developed [1]. Currently, pregnancy intentions are often measured retrospectively for research purposes, to indirectly assess contraceptive behavior and unmet need for contraception, estimate their association with maternal and child health outcomes, and evaluate programmatic efforts to reduce unintended pregnancy [2]. Survey items often limit pregnancy motivation into the classification of intended or unintended, with the latter further defined as mistimed or unwanted. Clinicians also use measures to gauge fertility intentions prospectively in clinical encounters, in an effort to determine patients’ need for contraception or preconception care [3, 4].

This intentions-oriented framework assumes that a planned approach to becoming pregnant or avoiding pregnancy is universal. It also presumes that perceptions about pregnancy fall into discrete categories of intended or unintended and does not account for those whose pregnancy desires may fall outside of these categories or along a continuum of intentions, such as those who may hold seemingly conflicting views about their control over and feelings regarding pregnancy [2, 5]. Research has focused on investigating how sociodemographic characteristics, such as race/ethnicity and income level, are associated with pregnancy intentions [6]. This does little, though, to help identify if and what areas of need exist, beyond labeling sub-groups as being “at risk” of unintended or unplanned pregnancies.

In turn, much contraceptive-related research has narrowed its focus to women of color and those of low income, that is, those with the highest rates of unintended pregnancy [6–13]. Although these efforts are ostensibly intended to inform provision of health care services to those most in need of them, this focus could also be interpreted as problematizing pregnancies among certain sub-groups, particularly given the decades of reproductive coercion in the U.S. [14–17]. A more nuanced understanding, from research that includes women and men from a range of socioeconomic and racial/ethnic backgrounds, may inform development of a more comprehensive, valid framework of pregnancy-related desires and beliefs, and an improved understanding of related behaviors.

Researchers have challenged the current approach of measuring pregnancy intentions [18, 19]. A recent body of qualitative research suggests that commonly used measures of pregnancy intentions do not always align with how people conceptualize and approach pregnancy [2, 18, 20–23]. Experts in the field suggest a redirection, to assess other factors such as perceptions of and emotions associated with pregnancy [18] and pregnancy “acceptability” [24]. There is also a call to differentiate how people think about avoiding pregnancy and becoming pregnant, as separate constructs [5].

Research that has examined those with “ambivalent” feelings about a pregnancy have found that this group is less likely to use contraception consistently and more likely to use less effective methods [25, 26]. Another study investigated the attitudes, social pressures, and sociodemographic characteristics of women trying to become pregnant, not trying, or okay either way. The authors found that for many attitudinal and social pressure variables, such as importance of career and a child being important to partner, those “okay either way” scored between those who were and were not trying to become pregnant [27]. Although newer measures of pregnancy have incorporated notions of happiness, ambivalence, desires, emotions, consequences, and relationships [5, 25, 28, 29], it remains unclear how emotions and various perceptions of pregnancy are related to different levels of pregnancy desires, beyond the simplistic binary assessment of “intended” and “unintended.”

As our understanding of how people think about and approach pregnancy continues to evolve, so should our conceptualization and measurement of the construct. Qualitative research suggests that women and men think about pregnancy in a way that is deliberate (planned), predetermined (‘just happens’ or left to fate), or a blend of both [22, 23]. As a next step, using this framework in a survey-based approach, and to fill the gaps identified above, we sought to gain a deeper understanding of how such multidimensional beliefs about pregnancy are associated with pregnancy desires; our analysis contextualizes the experiences and beliefs of women and men from a range of racial/ethnic and socioeconomic backgrounds, differentiates how people think about avoiding pregnancy and becoming pregnant, and assesses emotions related to different levels of pregnancy desires.

Specifically, we sought to examine if and how beliefs about avoiding pregnancy and becoming pregnant, and perceived control over pregnancy, are associated with *wanting a pregnancy*, *not wanting a pregnancy*, and *not trying but feeling okay about a pregnancy*. This approach captures those who may not fall squarely into the categories of having an “intended” or “unintended” pregnancy; that is, those *not trying to become pregnant but who would be okay with a pregnancy* [27]. To understand more about what is driving the seemingly inconsistent and ineffective use of contraception among those with “ambivalent” feelings towards pregnancy [25, 26], we also investigated how consistent contraceptive use among those *not trying but okay with a pregnancy* varies by different beliefs and emotions. We hypothesized that those who had beliefs about avoiding pregnancy and becoming pregnant that fell into the “predetermined” group would be more likely to report *not trying but would be okay with becoming pregnant soon*, versus *not wanting to become pregnant soon*. We also hypothesized that, among those who reported *not trying but would be okay with becoming pregnant soon*, consistent contraceptive use would be associated with negative emotions about having a pregnancy soon and “deliberate” perceptions about pregnancy. Our findings can help advance our understanding of people’s pregnancy desires, feelings, and beliefs, to ultimately inform newer valid measures that could better identify contraceptive or preconception counseling needs.

## Methods

### Study design & recruitment

We used non-probability quota-based sampling according to the 2010 Census, applying national rates of sex, age (in 5-year categories from age 21–44), and the four regions of the United States (U.S.) to inform the sampling approach. Respondents needed to read and understand English to participate. Our aim was to explore beliefs about pregnancy among young and older adults, thus we included participants starting at age 21. Our study was based on formative research which did not include younger adolescents, whose beliefs towards pregnancy are likely distinct (given their younger age/different life experiences) and would have required different survey questions [23]. We also excluded those who self-identified as gay or lesbian who would also require different survey items and be beyond the scope of this study.

A third-party, online recruiting firm (Qualtrics^™^) handled sampling and monitored enrollment until established rates within each category were met. We administered a cross-sectional web-based survey of heterosexual or bisexual men and women, living in the U.S., to explore how pregnancy desires were associated with emotions related to a pregnancy ‘right now,’ control over becoming pregnant or avoiding pregnancy, and beliefs about avoiding and becoming pregnant. The response rate was approximately 19%, with a total of 2,099 respondents who completed the survey. We determined a target sample size (power = 0.95; alpha = 0.05) of at least 233 respondents in each outcome category (mistimed, unwanted, or intended pregnancy- proxies for pregnancy desires used in this study) for analyzing associations with pregnancy happiness (proxy for feelings about a pregnancy) [30]. Participants were screened and consented before participation and received the incentive payment for participation via their survey panel membership.

The primary analysis examined factors associated with pregnancy desires. As such, we restricted the sample to those who could get pregnant, were not (or a sexual partner was not) currently pregnant, and responded in one of the three categories for the dependent variable of pregnancy desires (*would like to*, *do not want to*, or *are not trying but would be okay if became pregnant soon*; n = 1,477).

### Study instrument

Survey items were developed following an extensive review of the literature; cognitive interviews helped to refine item specificity and comprehension. Details of recruitment and survey instrument development are reported elsewhere [31]. The final survey included 45 questions related to: sociodemographic characteristics, ideal criteria before first pregnancy, current pregnancy desires, feelings related to a pregnancy ‘right now,’ control over avoiding pregnancy and becoming pregnant, beliefs about avoiding and becoming pregnant, and items related to thoughts about a first pregnancy (if applicable).

Pregnancy *desires* were assessed with the question “When thinking about your current situation, would you say that you/your partner: a) are pregnant, b) would like (your partner, or anyone you have sex with) to become pregnant soon, c) don’t want (your partner, or anyone you have sex with) to become pregnant soon, d) aren’t trying to become pregnant, but would feel okay about it if you (your partner, or anyone you have sex with) became pregnant soon, e) can’t get pregnant, or f) other)” (Table 1). Those who were pregnant, could not get pregnant or reported “other” were excluded from the analysis.

**Table 1:** Survey questions.

Domain	Item	Response options
Pregnancy desires	When thinking about your current situation, would you say that you/your partner…	a) are pregnant *(Excluded)* b) would like (your partner, or anyone you have sex with) to become pregnant soon c) don’t want (your partner, or anyone you have sex with) to become pregnant soon d) aren’t trying to become pregnant, but would feel okay about it if you (your partner, or anyone you have sex with) became pregnant soon e) can’t get pregnant *(Excluded)* f) other (specify) *(Excluded)*
Ideal criteria before pregnancy	Before (a partner) becoming pregnant, do you think there are ideal criteria that should be fulfilled first (things like finishing school, etc)?	a) yes b) no c) don’t know d) don’t ever want (a partner) to become pregnant
Feelings about a pregnancy	Ultimately, how would you feel about (a partner, or anyone you had sex with) being pregnant right now?	a) positive b) negative
Control over avoiding a pregnancy	How much control would you say you have over avoiding (a partner’s) pregnancy?	a) no control b) a little control c) a lot of control d) complete control
Control over becoming pregnant	If you wanted (a partner) to become pregnant, how much control would you say you have over (her) becoming pregnant?	a) no control b) a little control c) a lot of control d) complete control
Beliefs about avoiding pregnancy*	When thinking about trying to avoid (a partner) becoming pregnant, would you say that pregnancy…	a) can be avoided b) sometimes ‘just happens’ anyway c) is something determined by fate or a higher power like God d) is a natural process that happens when it’s meant to e) other (specify) *(Dropped)*
Beliefs about becoming pregnant*	When thinking about a pregnancy, would you say that it…	a) can be planned in advance b) can be planned to happen after one’s ideal criteria are fulfilled c) can be planned in discussion with your partner d) can be left to ‘fate’ or a higher power like God e) ‘just happens’ f) is a natural process that happens when it’s meant to be g) other (specify) *(Dropped)*

*Item was ‘select all that apply.’ If respondents chose more than one response to either beliefs questions, they were then asked to choose the one they “feel the most strongly about.”

*Feelings about a pregnancy* were measured with the question “Ultimately, how would you feel about (a partner, or anyone you had sex with) being pregnant right now? a) positive or b) negative.” We also asked respondents which, if any, of 11 listed emotions, they would feel if they or their partner was pregnant right now. *Control over avoiding pregnancy* was captured with the question “How much control would you say you have over avoiding (a partner’s) pregnancy?” and *control over becoming pregnant* was assessed with the item “If you wanted (a partner) to become pregnant, how much control would you say you have over (her) becoming pregnant?” Both items had the response categories: a) no control, b) a little control, c) a lot of control, or d) complete control.

Ideal criteria was assessed with the question “Before (a partner) becoming pregnant, do you think there are ideal criteria that should be fulfilled first (things like finishing school, etc)? a) yes, b) no, c) don’t know, or d) don’t ever want (a partner) to become pregnant.” A follow-up question asked about specific criteria the respondent wanted to meet before becoming pregnant, including starting a career, and being in a relationship (item not used in this analysis).

*Beliefs about avoiding pregnancy* were captured with the question “When thinking about trying to avoid (a partner) becoming pregnant, would you say that pregnancy: a) can be avoided, b) sometimes ‘just happens’ anyway, c) is something determined by fate or a higher power like God, d) is a natural process that happens when it’s meant to, and e) other.” *Beliefs about becoming pregnant* were assessed with a similar item that was asked after an introduction which noted that the next series of questions were about *becoming* pregnant. The item used was: “When thinking about a pregnancy, would you say that it…a) can be planned in advance, b) can be planned to happen after one’s ideal criteria are fulfilled, c) can be planned in discussion with your partner, d) can be left to ‘fate’ or a higher power like God, e) ‘just happens,’ f) is a natural process that happens when it’s meant to be, and g) other.” If respondents chose more than one response to either beliefs questions, they were then asked to choose the one they “feel the most strongly about.” For this primary analysis we used their most strongly held belief about becoming pregnant and avoiding pregnancy (dropping those who chose “other”).

Among the sub-sample of those *not trying but would be okay with a pregnancy*, we also asked about pregnancy-related behavior with the item “You said you aren’t trying to become pregnant, but would feel okay if you (your partner, or anyone you have sex with,) became pregnant. Are you…a) just letting it happen, b) leaving it up to fate or a higher power like God, c) leaving it up to nature, d) using some form of birth control or contraception every time you have sex, e) using some form of birth control or contraception some of the time when you have sex, f) other (specify).” Respondents could choose all that applied.

### Statistical analysis

Bivariate analyses (chi square and fisher’s exact tests) and multinomial logistic regression explored the relationship between the main dependent variable of pregnancy desires (*want pregnancy*, *do not want pregnancy*, and *aren’t trying but would be okay about becoming pregnant soon*) with sociodemographic characteristics and ideal criteria, feelings, control, and beliefs related to pregnancy. Given the exploratory nature of this inquiry, we examined all comparison group pairs (*want pregnancy* vs. *do not want pregnancy*; *are not trying but would be okay with pregnancy* vs. *do not want pregnancy*; *are not trying but would be okay with pregnancy* vs. *want pregnancy*). As our aim was to understand more about the group that does not fit strictly into the binary pregnancy intentions measure, we conducted a sub-group analysis of those who responded that they *are not trying but would be okay with becoming pregnant soon* (n = 460) to assess how positive and negative feelings about a hypothetical pregnancy compare with individual emotions. In addition, using univariate statistics and chi-squared tests we examined how contraceptive use every time they had sex was associated with emotions related to a pregnancy ‘right now’ and beliefs about avoiding a pregnancy.

We used SAS^®^ version 9.4 for all statistical analyses. The study was approved by the Institutional Review Board (Protocol #2016–1013) of the CUNY Graduate School of Public Health and Health Policy, New York.

## Results

We present the results as follows. First, for the primary analysis examining how sociodemographic characteristics and ideal criteria, feelings, control over, and beliefs related to pregnancy are associated with pregnancy desires, we present univariate and bivariate statistics, followed by multinomial regression results. Next, we review the univariate findings on emotions among those who report *not trying but would be okay with becoming pregnant*. Lastly, we report chi-squared test results of this sub-group, examining the association between pregnancy-related feelings and beliefs about avoiding pregnancy, with consistent contraceptive use.

### Associations between pregnancy-related desires and perceptions

#### Univariate and bivariate results

Among the 1,477 respondents, almost half (49%) did not have children (not reported). The sample was 51% female. Most were white (63%), followed by Latinx (15%), and the largest proportion was married (40%). The sample was well distributed across age, sex, region, income, and educational categories, although few (3%) had less than a high school education.

Almost half (49%) reported that they *do not want to become pregnant soon* while 20% reported that they *would like to become pregnant soon* and almost one third of respondents (31%) reported that they were *not trying but would be okay becoming pregnant soon* (Table 2). Males and females were equally likely to report *not wanting to become pregnant soon*. However, such opinions diverged when asked about other pregnancy desires. More females (33%) reported *not trying but being okay becoming pregnant soon*, compared to males (29%; p<0.05); conversely, a higher percentage of males (22%) reported that they *would like a pregnancy soon* compared to females (17%; p<0.05). For single (unpartnered) respondents, a majority (61%) reported *not wanting a pregnancy soon*; although approximately one quarter (26%) reported *not trying but feeling okay if they became pregnant soon*.

**Table 2:** Univariate and bivariate results: Factors associated with pregnancy desires.

Variable	Total	Pregnancy Desires n (%)		
	n = 1,477	Would like (partner) to become pregnant soon	Aren’t trying, but would feel okay about (partner) becoming pregnant soon	Don’t want (partner) to become pregnant soon
	n (%)	n = 291 (20)	n = 460 (31)	n = 726 (49)
**Has ideal criteria before pregnancy**^†^				
Yes	1161 (79)	239 (21)	351 (30)	571 (49)
No	190 (13)	40 (21)	72 (38)	78 (41)
Don’t know	77 (5)	12 (16)	34 (44)	31 (40)
Don’t ever want (partner) to be pregnant	49 (3)	0 (0)	3 (6)	46 (94)
**Feelings about a pregnancy right now**^†^				
Positive	941 (64)	272 (29)	393 (42)	276 (29)
Negative	536 (36)	19 (4)	67 (12)	450 (84)
**Control over avoiding pregnancy**^†^				
Complete/A lot	1263 (85)	225 (18)	393 (31)	645 (51)
Little/None	214 (15)	66 (31)	67 (31)	81 (38)
Control over becoming pregnant (if wanted pregnancy)*				
Complete/A lot	1031 (70)	199 (19)	302 (29)	530 (51)
Little/None	446 (30)	92 (21)	158 (35)	196 (44)
**Beliefs about avoiding pregnancy**^†^				
Can be avoided	922 (65)	134 (14)	255 (28)	533 (58)
Sometimes ‘just happens’	223 (15)	54 (24)	68 (30)	101 (45)
Determined by fate or higher power	141 (10)	45 (32)	56 (40)	40 (28)
Natural process that happens when it’s meant to	140 (10)	47 (34)	57 (41)	36 (26)
**Beliefs about becoming pregnant**^†^				
Can be planned	466 (32)	98 (21)	121 (26)	247 (53)
Can be planned after ideal criteria are met	295 (20)	42 (14)	89 (30)	164 (56)
Can be planned in discussion with partner	415 (28)	70 (17)	138 (33)	207 (50)
Left to fate or higher power	108 (7)	32 (30)	39 (36)	37 (34)
‘Just happens’	53 (4)	14 (29)	17 (32)	22 (42)
Natural process that happens when meant to be	126 (9)	30 (24)	55 (44)	41 (33)

* p<0.05;

^†^p<0.01

Feelings about a pregnancy were diverse. While 36% reported having *negative* feelings about a pregnancy in the near future, 12% of this sub-group reported *not trying but being okay becoming pregnant soon* in response to the pregnancy desire question. In contrast, 29% of those who would feel *positive* about a pregnancy ‘right now’ said that they *did not want a pregnancy soon*.

Respondents also reported mixed opinions on both perceptions about avoiding pregnancy and becoming pregnant. In response to the question regarding beliefs about avoiding a pregnancy, over a third (35% total) of respondents reported their strongest belief to be that pregnancy could not be avoided, as it either sometimes ‘just happens’ (15%), is determined by fate or higher power (10%), or happens as a natural process when it is meant to happen (10%). In comparison, in response to the question regarding becoming pregnant, 80% reported that pregnancy could be planned, either unconditionally (32%), after ideal criteria are met (20%) or in discussion with a partner (28%).

#### Multinomial logistic regression results

Table 3 reports adjusted odds ratios (aOR) and corresponding 95% confidence intervals (CI) and p-values from the multinomial logistic regression model estimating the association between individual characteristics, ideal criteria, feelings about, control over, and beliefs related to pregnancy. The model adjusted for sex, number of children, age, relationship status, partner ability to conceive, geographic region, education, and race/ethnicity. Feeling negative about a pregnancy ‘right now’ was associated with lower odds of reporting *not trying but would be okay with a pregnancy* (aOR: 0.11, CI: 0.08–0.15), compared to those who *did not want a pregnancy*, but higher odds of reporting *being okay with a pregnancy* compared to those who *want a pregnancy* (aOR: 1.92, CI: 1.09–3.39).

**Table 3:** Multinomial model: Factors associated with pregnancy desires**.

Variable	Pregnancy Desires					
	Would like (partner) to become pregnant soon *(vs*. *Don’t want pregnancy)*		Aren’t trying, but would feel okay about (partner) becoming pregnant soon *(vs*. *Don’t want pregnancy)*		Aren’t trying, but would feel okay about (partner) becoming pregnant soon *(vs*. *Would like pregnancy)*	
	adjusted Odds Ratio (aOR)	95% Confidence Interval (CI)	adjusted Odds Ratio (aOR)	95% Confidence Interval (CI)	adjusted Odds Ratio (aOR)	95% Confidence Interval (CI)
**Has ideal criteria before pregnancy**						
Yes	1.0		1.0		1.0	
No	0.90	0.54–1.50	1.15	0.75–1.74	1.27	0.80–2.03
Don’t Know	1.36	0.59–3.14	1.85	0.99–3.47	1.36	0.64–2.93
Don’t ever want (partner) to be pregnant	< .001	<0.001->999.99	0.26*	0.07–0.94	>999.99	<0.001->999.99
**Feelings about a pregnancy right now**						
Positive	1.0		1.0		1.0	
Negative	0.06^†^	0.03–0.09	0.11^†^	0.08–0.15	1.92*	1.09–3.39
**Control over avoiding pregnancy**						
Complete/A lot	1.0		1.0		1.0	
Little/None	1.73*	1.03–2.92	1.09	0.68–1.74	0.63	0.39–1.01
**Control over becoming pregnant (if wanted pregnancy)**						
Complete/A lot	1.0		1.0		1.0	
Little/None	0.99	0.66–1.48	1.33	0.96–1.85	1.35	0.92–1.99
**Beliefs about avoiding pregnancy**						
Can be avoided	1.0		1.0		1.0	
Sometimes ‘just happens’	1.87*	1.16–3.02	1.21	0.80–1.83	0.65	0.41–1.02
Determined by fate or higher power	2.74^†^	1.44–5.22	1.83*	1.05–3.36	0.67	0.38–1.17
Natural process that happens when it’s meant to	3.03^†^	1.68–5.46	2.21^†^	1.29–3.76	0.73	0.44–1.22
**Beliefs about becoming pregnant**						
Can be planned	1.0		1.0		1.0	
Can be planned after ideal criteria are met	0.54*	0.33–0.91	1.18	0.79–1.76	2.17^†^	1.32–3.55
Can be planned in discussion with partner	0.69	0.44–1.07	1.12	0.77–1.60	1.61*	1.06–2.47
Left to fate or higher power	1.00	0.47–2.13	1.18	0.60–2.32	1.18	0.59–2.33
‘Just happens’	1.03	0.43–2.50	0.92	0.41–2.05	0.89	0.38–2.06
Natural process that happens when meant to be	0.77	0.38–1.53	1.24	0.68–2.23	1.61	0.87–2.98

**adjusted for sex, number of children, age, relationship status, partner ability to conceive, geographic region, education, and race/ethnicity

* p<0.05;

^†^p<0.01

In relation to beliefs about avoiding pregnancy, those who felt that pregnancy may not always be avoidable, but instead determined by fate/higher power or was a natural process, were significantly more likely to report *not trying but being okay with becoming pregnant soon* (aOR: 1.83, CI: 1.05–3.36; aOR: 2.21, CI: 1.29–3.76, respectively), compared to those who *did not want to become pregnant soon*.

In terms of beliefs about becoming pregnant, those who felt pregnancy can be planned after ideal criteria are met or in discussion with a partner were more likely to report *not trying but being okay with a pregnancy soon* (aOR: 2.17, CI:1.32–3.55; aOR: 1.61, CI: 1.06–2.47, respectively), compared to those who *want a pregnancy soon*. Remarkably, some of the demographic markers typically associated with differences in pregnancy ‘intendedness’ (such as race/ethnicity and education) were not associated with significant differences in pregnancy desires (not shown).

### Sub-group analysis among those not trying but are okay with pregnancy

#### Emotions related to pregnancy

In Table 4, we compare feelings about a pregnancy ‘right now’ to individual emotions among those *not trying but okay with becoming pregnant*. Interestingly, only 12 respondents (3% of this sub-group) reported feeling ambivalent about a pregnancy, and, of those, almost all reported that they would ultimately feel positive about a pregnancy. Most in this sub-group would feel excited (57%), surprised (53%), and/or happy (53%) about a pregnancy ‘right now.’ The majority of self-reported emotions mapped onto their expected overall feelings; for example, most of those who would be angry reported feeling negative and most who felt they would be thankful reported feeling positive about a pregnancy. However, the majority of those who felt frustrated, worried, or scared also reported feeling positive about a pregnancy. Despite feeling happy, excited, or thankful, a small portion of those respondents also reported that they would feel negative about a pregnancy ‘right now.’

**Table 4:** Pregnancy-related emotions among those not trying but would be okay with pregnancy (n = 460).

Emotion	Total	Feelings about a pregnancy right now	
		Positive	Negative
	n (%)	n (%)	n (%)
		n = 393 (85)	n = 67 (15)
Angry	10 (2)	1 (10)	9 (90)
Frustrated	21 (5)	11 (52)	10 (48)
Unhappy	8 (2)	1 (13)	7 (87)
Anxious	181 (39)	149 (82)	32 (18)
Worried	123 (27)	87 (71)	36 (29)
Scared	158 (34)	125 (79)	33 (21)
Surprised	243 (53)	216 (89)	27 (11)
Ambivalent	12 (3)	11 (92)	1 (8)
Happy	243 (53)	232 (95)	11 (5)
Excited	260 (57)	242 (93)	18 (7)
Thankful	155 (34)	149 (96)	6 (4)

#### Consistent contraceptive use

In Table 5 we present how beliefs about avoiding a pregnancy and feelings related to a pregnancy ‘right now’ are associated with consistent contraceptive use among those who *are not trying but would be okay with becoming pregnant soon* (n = 460). We did not find significant differences between those who reported positive or negative feelings about a pregnancy ‘right now’ with reports of consistent contraceptive use. When we examined attitudes regarding control over avoiding a pregnancy, there was a significant association with consistent contraceptive use (p<0.0001); those who felt pregnancy can be avoided were more likely to report using contraceptives with every sexual act (61%), compared to 39% who did not use contraception consistently. Interestingly, of those who believed pregnancy sometimes ‘just happens,’ there were about equal proportions who did and did not use contraception at every sexual encounter. Higher proportions of those who felt pregnancy was determined by fate or a higher power or a natural process also reported *not* using contraception every time one has sex (70%, 68%, respectively).

**Table 5:** Contraceptive use among those not trying but would be okay with pregnancy (n = 460).

Variable	Total	Contraceptive use every time one has sex	
		n (%)	
	n (%)	Yes	No
		n = 233 (51)	n = 227 (49)
**Feelings about a pregnancy right now**			
Positive	393 (85)	195 (50)	198 (50)
Negative	67 (15)	38 (57)	29 (43)
**Beliefs about avoiding pregnancy**^†^			
Can be avoided	255 (59)	156 (61)	99 (39)
Sometimes ‘just happens’	68 (16)	33 (49)	35 (51)
Determined by fate or higher power	56 (13)	17 (30)	39 (70)
Natural process that happens when it’s meant to be	57 (13)	18 (32)	39 (68)

^†^p<0.0001

## Discussion

The pregnancy-related measures used in this study move beyond the narrow focus on how some sociodemographic characteristics are associated with binary pregnancy intentions. Similar to recent research, by separating out those who are *not trying to become pregnant but would be okay with becoming pregnant soon*, we present an expanded picture of a pregnancy intentions or desires “continuum” [5, 27]. Moreover, operationalizing categories in this way may account for a proportion of individuals in past research who reported not having ‘intended’ to become pregnant but who were also not using contraception consistently or at all [32].

Perceptions about avoiding a pregnancy, in feeling that it is determined by fate or nature, are particularly salient for those who were *not trying but would feel okay about an imminent pregnancy*. The belief that pregnancies are predetermined in this way can explain why this group may not fit squarely into measures of pregnancy intentions. This was also reflected in their contraceptive behavior in that nearly half of those who are *not explicitly trying to become pregnant* noted not using contraception consistently. Those who felt pregnancy was determined by fate or nature were less likely to use contraception consistently, indicating that beliefs about avoiding pregnancy may have an impact on contraceptive behavior. This may explain the inconsistent contraceptive use and use of less effective methods found in other studies among those who were “ambivalent” about pregnancy [25, 26]. This approach to pregnancy may be an indication that people do not necessarily want to state an explicit desire to become pregnant [33]. Additionally, most of those who *aren’t trying but okay with pregnancy* ultimately felt positive about an imminent pregnancy. Deviating from longstanding conclusions regarding pregnancy intendedness and contraceptive use, our findings demonstrate that inconsistent contraceptive use and predetermined beliefs about pregnancy can be legitimate and acceptable, if this approach to pregnancy supports an individual’s or couple’s own pregnancy desires. Moreover, making assumptions regarding need for contraceptive counseling or education risks missing a different approach some people may take to ‘allow’ for a pregnancy to occur (i.e., one third of our sample expressed beliefs that pregnancy is predetermined). Counseling can be a useful tool to help support patients’ reproductive desires and autonomy, but if approached from the perspective of disabusing people of beliefs in order to improve contraceptive use, this may not be effective nor patient-centered. Further research should investigate if and how contraceptive counseling is wanted among those at different points along the pregnancy desires “continuum.”

Our findings that almost a third of those who felt positively about a pregnancy ‘right now’ also reported not wanting a pregnancy soon reinforce the notion that holding seemingly contradictory positions related to pregnancy is common [33]. It also suggests that emotions may not be the best indicator by which to assess pregnancy desires [34].

This study builds on the findings from research focused on pregnancy ambivalence. McQuillan et al studied women’s attitudes (around motherhood and religiosity), social pressures, and other characteristics among those who were “okay either way” about pregnancy. Our work adds to this by investigating how nuanced beliefs about, perceived control over, ideal criteria, and emotions related to pregnancy differ among those who are “okay either way” with a pregnancy; our study also included men’s perspectives [27]. A recent study of young couples by Gomez et al demonstrated that the many ways in which research has classified pregnancy intentions as ambivalent “…fail to capture the intricacies of pregnancy intentions…” [35] Our findings also document the complexities of the pregnancy intentions classification; only 3% of those who were *not trying but okay with a pregnancy* reported feeling ambivalent about a pregnancy. As researchers, we may have assumed ambivalence and *not trying but being okay with a pregnancy* to be the same constructs, but our data suggest otherwise.

Notably, once we accounted for perceptions related to pregnancy, individuals’ race/ethnicity, and education were unassociated with pregnancy desires. This suggests a redirection of public health efforts from focusing on subgroups by sociodemographic factors, such as race/ethnicity, in order to reduce unintended pregnancy, to instead exploring beliefs related to pregnancy, and how such beliefs are associated with contraceptive-related behaviors.

Although pursuing research to understand more about beliefs of how pregnancy happens can help guide public health efforts, this approach remains rooted in an individual-level model of health behavior. Individual beliefs and behaviors are embedded within one’s larger social, economic, and political constraints and research on contraception and reproduction should reflect as such. Thus, incorporating more macro-level measures and determinants into this work would be valuable. Some have suggested measuring access to contraception and abortion, as indicators of reproductive autonomy [19]. Potter et al suggest querying women and men about not only the contraceptive method they are using, but what they would ideally like to be using, as a means of evaluating if they have access to their preferred method. MacLeod also suggests we reconsider intentions altogether and instead measure pregnancy “supportability” [36]. Her model highlights the micro- and macro-level supports available or lacking for *having* a pregnancy; researchers and policy makers can expand this framework to include *preventing* a pregnancy. Similarly, in the clinical encounter, instead of asking about fertility intentions, research suggests that patients and primary care physicians both prefer incorporating an open-ended question that offers help in providing non-specific reproductive health services [37, 38]. This patient-centered approach can effectively avoid the pitfalls of predicting fertility.

This study has limitations in that we cannot assess if beliefs and perceptions about pregnancy lead to pregnancy desires or vice versa. Although we have representation across all regions of the U.S., given that this was a non-probability sample it is not representative of the general population. Individuals may have interpreted items differently, such as what it means for pregnancy to be a “natural” process. To minimize this threat to validity, however, we used language in the survey that we adopted from our previous qualitative work of nearly 200 men and women, and conducted cognitive interviews to improve question clarity and interpretation. We also asked about prospective intentions and feelings about a hypothetical pregnancy which may not ultimately align with respondents’ experience with a real pregnancy. The study sample was diverse on a number of key demographic characteristics. As such, this analysis offers a more nuanced examination of individuals with a wide range of characteristics situated along the continuum of pregnancy desires and identifies important factors and behaviors associated with such desires.

Our analysis builds on the foundation of qualitative work and adds a more complex examination of how emotions and beliefs related to pregnancy are associated with pregnancy desires, particularly among those who are *not trying but would be okay with a pregnancy*. This work informs a deeper understanding of this phenomenon, identifying future research paths and pointing to areas for providing support to help all individuals meet their pregnancy desires. Perspectives on pregnancy are more complex than current intentions-based measurements capture. Findings from our study provide credence to claims of the complexity of beliefs about pregnancy that are not adequately captured with pregnancy planning or intentions-oriented frameworks [18, 19, 39]. Individuals hold a spectrum of beliefs about their ability to avoid pregnancy and become pregnant, which ultimately have implications for reproductive health service provision and contraceptive use. Further use and development of measures that capture the complexity surrounding pregnancy, such as those we employed in this study, are likely to produce more valid findings regarding Americans’ fertility-related attitudes and behaviors.
